# The impact of clinical context on the recognition of facial expressions

**DOI:** 10.1192/j.eurpsy.2024.271

**Published:** 2024-08-27

**Authors:** C. De Sousa, S. Morgado, J. Ferreira, S. Tukaiev, R. Fonseca

**Affiliations:** ^1^Higher School of Health Atlântica, Barcarena; ^2^ISEIT, Almada; ^3^Faculty of Medicine, Coimbra University, Coimbra, Portugal; ^4^Università della Svizzera italiana, Lugano, Switzerland

## Abstract

**Introduction:**

Several authors have demonstrated the relevance of the therapist sensitivity to the affective expression of his client (Merten & Schwab, 2005; 150-158), as well as to his own emotional experience (Haynal-Raymond et al., 2005;142-148) in order to build a more effective therapeutic relationship, and results. An important source of information to decode the emotional expression hints is the face, and its expression (Ekman & Friesen, 1975; Russel & Fernández-Dolls, 1997;275-294). Despite common sense saying that context is relevant to understand the meaning of the emotional facial expression, the literature review shows inconsistent results.

**Objectives:**

The main goal of this study was to evaluate the impact of clinical context over the perception of the emotional facial expression.

**Methods:**

This study followed a within-subjects design, and its sample consisted of 60 clinical psychologists. 21 combinations of prototypical expression images with mixed emotional signals, and clinical information texts were presented to the participants. Then their judgement on the type of emotion displayed was requested. The presentation of the text-image pairs was randomized between three conditions: consistent, and non-consistent, and neutral.

**Results:**

The results suggest that emotions are more easily recognized in the presence of a concordant context than a non-concordant or neutral one, and that the greater the similarity between the facial expression of the image presented and the face prototypically associated with the context, the greater the influence of the context.

However, In the recognition of mixed emotional signs, there was greater recognition of signs of anger in the facial expression, as a non-dominant emotion, when in the presence of the neutral story than of the story that agreed with the dominant emotion (sadness). There was also greater recognition of sadness, as a non-dominant emotion, in the presence of a story in agreement with fear than in the presence of a neutral story. There was also a statistically significant increase in the attribution of anger to images in which it is not present and whose dominant emotion is fear, when associated with a context of aggression vs. a neutral context.

It was also found that there was a significant decrease in the attribution of fear to the sadness-anger image (25%-75%) in the presence of the aggression context compared to the neutral and panic contexts.There was also a statistically significant decrease in the attribution of sadness to an image of fear in the neutral context compared to the other contexts (panic and aggression).

**Conclusions:**

In conclusion, our study have shown an impact of context over overvaluation or the undervaluation of the emotional facial expression as well as either with prototypical expressions or the mixed emotional signals when referring to sadness, fear, and anger. Thus, mental health clinicians should consider the influence of these contexts.

**Disclosure of Interest:**

None Declared

